# 

**Published:** 1900-02

**Authors:** 


### PERSONALS.

Among those present at the annual banquet of the College Veterinary Society of the University of Pennsylvania in December were to be noted Drs. Adams, Harger, Harshberger, and Hoskins, of the teaching staff, and Dr. Seeley, of the graduates.

Dr. W. B. E. Miller, recently of the Garfield Quarantine Station of New Jersey, has been transferred to the Philadelphia inspection department.

Dr. William J. Finn, of Brooklyn, conducts the Long Island Veterinary Hospital in conjunction with his outdoor practice.

Dr. D. B. Dennish, of Morrisville, Pa., was a visitor to the Journal office in January. He reported a very much improved condition in veterinary practice during 1899.

Dr. J. W. Dunham, of Fargo, North Dakota, fills the rôle of chief veterinarian to North Dakota and veterinarian to the State Agricultural College.

Dr. M. H. Reynolds, of Minneapolis, Minn., addressed the State Agricultural Society at St. Paul in January on "State Work with Infectious Diseases of Animals."

Dr. J. H. Luff, of Hudson, N. Y., makes a specialty of diseases of the respiratory organs in veterinary practice.

Drs. Leonard Pearson, of Philadelphia, and H. D. Gill, of New York, were the guests of Dr. Runge, of Newark, N. J., on a visit in January to the Francisco dairy at that point.

Dr. J. Stewart McClure, of Oxford, Pa., has been appointed assistant State veterinarian of Montana.

Dr. D. F. Luckey, of Columbia, Mo., has succeeded Dr. T. E. White as State veterinarian of that State.

Dr. Leonard Pearson addressed in January the State Breeders' Association on "Sanitary Stables" at Pittsburg.

Dr. C. M. Teegarden, of Warsaw, Ind., who has not been in active practice for some years, is about to resume professional duties again.

Dr. E. C. Fox, of Baltimore, Md., is fitting up a hospital to be used in connection with his outdoor practice.

Dr. L. D. Horner, of Woodstown, N. J., was a visitor to Philadelphia in January, reporting practice as unusually good in his district.

Dr. John F. McAnulty, of Philadelphia, has been on the sick-list for some time.

Dr. E. C. Fox, of Baltimore, Md., was a visitor to Philadelphia in January.

Dr. Leonard Pearson, of Philadelphia, addressed the Bradford County Farmers' Institute at Canton in December.

Dr. W. E. Kinney, of Madison, South Dakota, combines the dealing in real estate with his professional vocation.

Dr. G. W. Pope, of Boston, succeeded Dr. W. B. E. Miller at the Garfield Quarantine Station, New Jersey.

Dr. Abraham L. Lehman, of Mullen, Montana, assistant State veterinarian, has relinquished his post of duty in this direction to enter upon the practice of human medicine.

Dr. W. Horace Hoskins, of Philadelphia, was a visitor to Harrisburg in January in conference with Governor Stone relative to matters pertaining to the State Board of Veterinary Medical Examiners.

Dr. Wm. H. Welch, of Johns Hopkins University of Baltimore, is much interested in the study of the pathology of osteoporosis in horses.

Governor Stone, of Pennsylvania, reappointed Dr. James W. Sallade, of Pottsville, a member of the State Board of Examiners in January.

Dr. John J. Repp, of Ames, Iowa, is one of a committee of three of the State Association to seek legislation in the interest of the profession in the "Hawkeye State."

Dr. Jacob Helmer, of Scranton, Pa., was a visitor to several of the Eastern cities of Pennsylvania in January, investigating the milk-inspection systems and regulations.

Dr. F. H. Mackie, of Fairhill, Md., is a member of the State Legislature.

Drs. Leonard Pearson and M. E. Conard addressed the meeting of the State Board of Agriculture at Harrisburg, Pa., in January.

Dr. James A. Marshall, of Philadelphia, was unanimously elected Vice-President of the Philadephia Turf Club. Dr. Marshall takes a keen interest in all that pertains to the development of the American trotter.

Dr. M. E. Conard, of West Grove, Pa., was made a member of the Executive and Legislative Committees of the State Agricultural Society at its annual meeting in January.

Dr. W. W. Gardiner, of Hughesvile, Pa., has returned to his native heath, and is now located at Mt. Laurel, N. J.

Dr. Harri Dell, of Wabash, Ind., has received an appointment in the inspection service of the Bureau of Animal Industry and directed to report at South Omaha, Neb., for duty.

Dr. Chas. E. Bridge, of West Philadelphia, met with an accident in December by being struck by a trolley car.

Dr. T. E. Smith, of Jersey City, N. J., holds the office of veterinary surgeon to the Board of Police Commissioners of that city.

Dr. M. H. Henderson, of Syracuse, N. Y., holds the position of State veterinarian for the entire Commonwealth.

Dr. W. H. Dalrymple, of Baton Rouge, La., who is Secretary of the Louisiana State Agricultural Society and Stockbreeders' Association, presented an address before their meeting at Arcadia on January 25th on "The Value of Artificial Immunization Against Texas Fever to the Cattle Interests of the State."

Dr. Brimhall, field veterinarian to the Minnesota State Board of Health, has been taking a well-earned vacation, devoting his spare time to a course of special pathology at the State University.

Dr. Reynolds gave a lecture on the "Physiology of Digestion" to special dairy students in the School of Agriculture on January

8th; presented an annual report as Director of the Veterinary Department at the State Board of Health meeting, January 9th; gave a paper on "State Work with Infectious Diseases of Animals" at the joint meeting of the State Agricultural Society and Minnesota Stockbreeders' Association, January 10th; presided at the meeting of the Minnesota State Veterinary Medical Association, January 11th and 12th, presenting a paper on "Recent Progress in Veterinary Surgery," gave the president's address; in charge of the Association clinic Thursday evening, January 11th, at the University Hospital. In addition to this he provided for two different classes daily for the week in the University, and declined, on the grounds of physical impossibility, invitations to take part in the programmes of the Tuberculosis Convention at Des Moines, January 9th, and the programme of the Iowa State Veterinary Association, January 10th and 11th, all occurring during one week.

Dr. A. F. Martin, formerly of Fishkill Landing, N. Y., is now in the inspection department of the Bureau of Animal Industry, and located at Boston, Mass.

Dr. W. F. Derr, of Wooster, Ohio, holds the position of live-stock inpector for the Live-stock Commissioners of Ohio and conducts a large infirmary in connection with his practice.

Dr. Wm. G. Shaw, recently stationed at the Bureau of Animal Industry, Kansas City, Mo., has been ordered to Nogales, Arizona, to take charge of that station for the importation of Mexican stock.

Dr. George A. Evans, of San Francisco, California, has been compelled to retire from active practice by the serious inroads of chronic rheumatism.

Dr. Beale A. Robinson, of Parsons, Kansas, is a matriculate at the Ontario Veterinary College, Toronto, Canada, the present session.

Dr. D. Coristine, of Maple Creek, Assinaboine, Northwest Territory, fills the rôle of Veterinary Staff Sergeant to the Northwest Mounted Police.

Dr. Chas. Gresswell, of Denver, Colo., addressed the National Live-stock Association in January on the subject of "Tuberculosis from the Range Standpoint."

Dr. George W. Turner, of Highwood, Ill., is very enthusiastic over the selection of Detroit for the meeting place of the American Veterinary Medical Association for 1900.

